# A Neoglycoconjugate Containing the Human Milk Sugar LNFPIII Drives Anti-Inflammatory Activation of Antigen Presenting Cells in a CD14 Dependent Pathway

**DOI:** 10.1371/journal.pone.0137495

**Published:** 2015-09-04

**Authors:** Smanla Tundup, Leena Srivastava, Thomas Norberg, Wendy Watford, Donald Harn

**Affiliations:** 1 Department of Infectious Diseases, College of Veterinary Medicine, University of Georgia, Athens, Georgia, 30602, United States of America; 2 Center for Tropical and Emerging Global Diseases, University of Georgia, Athens, Georgia, 30602, United States of America; 3 Department of Biochemistry and Organic Chemistry, Uppsala University, Uppsala, Sweden; 4 Department of Microbiology, University of Chicago, Chicago, Illinois, 60637, United States of America; The Ohio State University, UNITED STATES

## Abstract

The milk pentasaccharide LNFPIII has therapeutic action for metabolic and autoimmune diseases and prolongs transplant survival in mice when presented as a neoglycoconjugate. Within LNFPIII is the Lewis^x^ trisaccharide, expressed by many helminth parasites. In humans, LNFPIII is found in human milk and also known as stage-specific embryonic antigen-1. LNFPIII-NGC drives alternative activation of macrophages and dendritic cells *via* NFκB activation in a TLR4 dependent mechanism. However, the connection between LNFPIII-NGC activation of APCs, TLR4 signaling and subsequent MAP kinase signaling leading to anti-inflammatory activation of APCs remains unknown. In this study we determined that the innate receptor CD14 was essential for LNFPIII-NGC induction of both ERK and NFkB activation in APCs. Induction of ERK activation by LNFPIII-NGC was completely dependent on CD14/TLR4-Ras-Raf1/TPL2-MEK axis in bone marrow derived dendritic cells (BMDCs). In addition, LNFPIII-NGC preferentially induced the production of Th2 “favoring” chemokines CCL22 and matrix metalloprotease protein-9 in a CD14 dependent manner in BMDCs. In contrast, LNFPIII-NGC induces significantly lower levels of Th1 “favoring” chemokines, MIP1α, MIP1β and MIP-2 compared to levels in LPS stimulated cells. Interestingly, NGC of the identical human milk sugar LNnT, minus the alpha 1–3 linked fucose, failed to activate APCs via TLR4/MD2/CD14 receptor complex, suggesting that the alpha 1–3 linked fucose in LNFPIII and not on LNnT, is required for this process. Using specific chemical inhibitors of the MAPK pathway, we found that LNFPIII-NGC induction of CCL22, MMP9 and IL-10 production was dependent on ERK activation. Over all, this study suggests that LNFPIII-NGC utilizes CD14/TLR4-MAPK (ERK) axis in modulating APC activation to produce anti-inflammatory chemokines and cytokines in a manner distinct from that seen for the pro-inflammatory PAMP LPS. These pathways may explain the *in vivo* therapeutic effect of LNFPIII-NGC treatment for inflammation based diseases.

## Introduction

Ligation of innate receptors on antigen presenting cells (APCs) by pathogen/self molecules is a key factor in activation and maturation of APCs into cells that help direct the ensuing immune response. How different ligands can bind to the same innate receptor complex on APCs, yet induce disparate signaling and maturation pathways, remains a largely unknown process. In this regard, neo-glycoconjugates (NGC) of the human milk sugar LNFPIII (LNFPIII-NGC) have therapeutic activity *in viv*o to treat or prevent inflammation based diseases including suppressing the severity of experimental autoimmune encephalomyelitis (EAE) and CNS inflammation[1], improving insulin sensitivity and metabolic functions in obesity[2], and prolonging allogeneic heart transplant survival[2]. The LNFPIII pentasaccharide contains the Lewis^x^ motif, found on the surface of *S*. *mansoni* schistosomula and schistosome eggs[3]. Neo-glycoconjugates (NGC) of lacto-*N*-fucopentaose III (LNFPIII) contain multiple (8 to 12) LNFPIII residues linked to a 40kDa dextran as carrier molecule [4]).

The ability of LNFPIII-NGC to drive anti-inflammatory responses *in vivo* appears linked to LNFPIII induced maturation of IL-10 producing macrophages[2]. LNFPIII-NGC are internalized by APCs via a receptor mediated and clathrin dependent process, that results in alternative activation of APCs and Th2 immune responses[5]. Targeting innate immune cells for treatment of inflammation based diseases is a novel approach that is distinctly different from the majority of anti-inflammatory treatments that target T cells.

Clearly, LNFPIII-NGC activates and induces maturation of APCs that are functionally, polar opposite to APCs activated and induced by the TLR4 ligand LPS. Nonetheless, LNFPIII-NGC activation and maturation of dendritic cells that are anti-inflammatory and Th2 driving phenotype also occurs via a process requiring TLR4. In a controversial study, we observed that LNFPIII-NGC stimulated TLR4-/- DCs were unable to drive CD4^+^ T cells to produce IL-4[6]. The inability to drive CD4+ T cells to Th2-type was associated with defects in activation of MAPK and NFkB signaling pathways in TLR4-/- BMDCs, as measured by activation of ERK1/2 and nuclear translocation of NFkB p50[4, 6]. Thus we have a paradox; the apparent requirement of the pro-inflammatory TLR4 signaling pathway for LNFPIII-NGC to induce maturation of anti-inflammatory APCs.

In an attempt to resolve this paradox, we looked further into the TLR4 receptor complex, comprised nominally of TLR4, MD2 and CD14 as co-receptors to trigger signaling cascades for APC activation. CD14 is the first pattern recognition receptor described, and in addition to TLR4, has been shown to assist TLRs-3, -7 and 9 in mediating immune activation[7–9]. In the study reported here, we initially asked if the TLR4 receptor complex is sufficient or if additional innate receptors play roles in LNFPIII-NGC APC activation. We found that CD14 via the TLR4-Ras-Raf1-Syk-TPL2-MEK signaling cascade is essential for LNFPIII-NGC induced ERK activation. Interestingly, LNnT, the tetrasaccharide backbone of LNFPIII, failed to activate APCs via the TLR4/MD2/CD14 receptor complex, suggesting that the alpha 1–3 linked fucose in LNFPIII but not in LNnT is required for this pathway. In a CD14 and MAPK pathway dependent manner, LNFPIII-NGC stimulated APCs did not produce IL-6, IL-12 or TNF-α but did produce “Th2 favoring” chemokine CCL22 and IL-10. Further, using confocal microscopy, we demonstrate that LNFPIII-NGC co-localizes with CD14 on APCs. Over all, this study provides additional insight into the mechanism of LNFPIII-NGC induced immune activation of APCs. The results presented here are likely to further define new targets to improve therapy of autoimmune and inflammation based diseases.

## Materials and Methods

### Ethics statement

The guide for the Care and Use of Laboratory Animals was in accordance with American Association for Accreditation of Laboratory Animal Care (AALAC) as well as Animal Welfare Act (AWA) and other applicable federal and state guidelines. Mice were anesthetized using CO2 method followed by physical method of cervical dislocation to completely euthanize the mice. All animal work presented here was approved by Institutional Animal Care and Use Committee (IACUC) of University of Georgia (AUP No. A2009–04–086).

#### Mice

C57BL/6, *Cd14* ^*-/-*^, or *Tlr4* ^*-/-*^ mice were obtained from Jackson Laboratory. Mice were housed at the CVM Rodent Vivarium facility (University of Georgia) under specific pathogen free-conditions following institutional guidelines.

#### Cell culture

RAW 264.7 cells (ATCC) and HEK293 cells (ATCC), HEK-Blue™ hTLR4 (InvivoGen) were grown in Dulbecco’s Modified Eagle Medium (DMEM) (Hyclone) supplemented with 10% fetal calf serum (Atlanta Biotech), 100U/ml penicillin, 100ug/ml streptomycin (Hyclone) and 2mM Glutamate-L. Cells were plated in (12, 24 or 96 well plates) then cultured in a humidified incubator at 37°C with 5% CO2 until they reached 70% confluency.

Adherent peritoneal macrophages were obtained from the peritoneal cavities of naïve *wt* and *Cd14* ^*-/-*^ mice by saline lavage, followed by plastic adherence of peritoneal exudate cells for 2–4 hrs at 37°C. Then, the supernatants were discarded and the adherent cells collected and washed 3X with warm PBS, counted and used for subsequent assays.

Bone marrow derived macrophages (BMDMs) were obtained by flushing bone marrow cells from tibia and femurs with media and culturing them in Dulbecco’s modified Eagle medium supplemented with 10ng/ml of MCSF (PeproTech Rocky hill NJ) essentially as described [4]. Media was replaced every 2^nd^ day with fresh MCSF and cells were used on day 6. TPL2 deficient BMDMs were kindly provided by Dr. Wendy Watford (UGA).

Bone marrow derived Dendritic cells (BMDCs) were obtained by flushing bone marrow cells in RPMI 1640 and culturing the cells with 20ng/ml of GMCSF (PeproTech Rocky hill NJ) essentially as described [10]. On day 3 and 5 fresh media containing GMCSF were added to the cells. On day 7 non-adherent cells were used for experiments. Cells were >90% CD11c+ dendritic cells as determined by Flow Cytometry.

#### Antibodies

LNFPIII-NGC was stained with monoclonal antibody E.5 (IgM) that recognizes LNFPIII/Lewis^x^[11]. Primary and neutralizing antibodies for CD14 were purchased from BD, Biosciences. Antibodies against Raf1, pERK and total ERK were purchased from Cell Signaling, Beverly, MA. Anti-p50/p105 antibody was purchased from eBiosciences, CA. anti-MBP was purchased from UPSTATE, Millipore, MA. Anti-TFIID antibody was purchased from Santa Cruz, CA.

#### Chemicals and reagents

Chemical inhibitors against ERK, Raf1, Ras, Syk and Src were purchased from Calbiochem, San Diego, CA. Recombinant GM-CSF and M-CSF used for generating BMDCs and BMDMs were purchased from PeproTech, Rocky Hill, NJ. Transfecting reagent Lipofectamine was purchased from Invitrogen, NY, USA and Opti-MEM media from Gibco, NY, USA. LNFPIII-NGC was produced by Thomas Norberg. Briefly, LNFPIII purified from human milk was purchased from Dextra, United Kingdom, and 40 kDa Dextran was purchased from Sigma. Both were shipped to Dr. Norberg who prepared LNFPIII-Dextran NGC using proprietary linker-spacer and conjugation chemistry. On average LNFPIII-NGC had 10–11 LNFPIII monomers per each 40 kDa dextran, equaling approximately 17% of theMW of the LNFPIII-Dextran conjugate. Thus, for each 50ug/ml of LNFPIII-Dex added, only 8.8 ugs was LNFPIII.

#### Quantitative RT-PCR

Cells were lysed and total RNA was purified using RNeasy spin column (Qiagen). One microgram (1μg) of RNA was used to perform RT-PCR followed by qRT-PCR using primers (Applied Biosystems, Foster city, CA) against specific genes, Universal PCR master mix and run on an ABI StepOne Plus (Applied biosystems, Foster city, CA). Expression was normalized to GAPDH endogenous control and fold expression was measured by comparing to the expression of the uninfected controls.

#### ELISA

ELISA was performed to measure the production of cytokines in cell culture supernatants. For IL-6, IL-10, IL-12 and TNFα sandwich ELISA was performed according to the manufacturer’s instructions (BD Pharmingen, San Diego, CA).

#### Western blot

BMDCs, BMDMs or RAW 264.7 cells were stimulated with LNFPIII-NGC (50μg/ml), dextran career (50μg/ml) or LPS (100ng/ml) for various time points and cells were lysed in lysis buffer (Cell Signaling, Technology, MA, USA). Total protein was estimated using BCA protein estimation kit (Pierce Biotechnology, Rockford, IL) and fractionated using SDS-Gel electrophoresis followed by western blot analysis using antibody against phospho-ERK, total ERK1/2, p50 (Santa Cruz Biotechnology), TFIID, pMBP

#### Reporter assay

For NFkB reporter assay, HEK-Blue™ hTLR4 (InvivoGen) was incubated with LPS (100ng/ml), LNFPIII (50μg/ml), Dextran carrier (50μg/ml) or nothing for 24 hrs at 37°C. Supernatant was collected and alkaline phosphatase levels were measured by adding the substrate as per manufacturer’s instruction.

#### Raf1 kinase assay

The Raf1 kinase assay was adapted from Gringhuis et al [12]. BMDCs and RAW264.7 macrophages were stimulated with serum free media with LPS (100ng/ml), LNFPIII (50μg/ml), Dextran carrier (50μg/ml) or none. Cells were harvested and lysed for 30 minutes on ice in lysis buffer (Cell Signalling, CA), and lysates were prepared, incubated with anti-Raf1 antibody (1:100) for 1–2 hrs at 4°C then Protein G Sepharose was added at 1:10 dilution overnight. The immune complexes were centrifuged and washed twice with lysis buffer and once with assay buffer (50mM Tris-HCl, pH 7.5; 20mM MgCl2). The immune complexes were resuspended in Assay Buffer containing 1.5 mM DTT, 1mM ATP and 1 μg of inactive MEK1 (Santa Cruz) as substrate and incubated for 20 minutes at 30°C with agitation. The reaction was stopped by addition of Laemmli lysis buffer and boiling the complex for 5 minutes. The cell lysates were then run on SDS PAGE gel for western blot analysis of phosphorylated MBP (Upstate/Millipore) or ERK.

#### Immunofluorescence

Cells were fixed with freshly prepared 3% paraformaldehyde (Sigma Aldrich) for 10min at room temperature then permeabilized with 0.05% Triton X-100 in PBS. Fixed cells were blocked in goat serum or 10% BSA for 20 min at room temperature then incubated with primary antibodies followed by Alexa-488/594 conjugated secondary antibodies for 1 hr at room temperature. Cells were counterstained with Hoechst dye (blue) for nuclear visualization and mounted in ProLong Gold mounting media (Molecular Probes). Images were acquired using a Nikon Eclipse Ti A1R Confocal Microscope System under 60X objective.

#### SiRNA mediated knockdown of Raf-1

BMDCs were seeded in 12 well plates at 1x10^6^ per well density in RPMI (Hyclone) media. Next day cells were transfected with anti-Raf-1 siRNA or non-target control for 48 hrs. BMDCs were then stimulated with LNFPIII-NGC (50μg/ml), dextran career (50μg/ml) or LPS (100ng/ml) for 60 mins at 37°C and processed for western blot analysis.

#### Statistical analysis

Statistical analyses were performed using Prism (GraphPad Software) and *p* values were obtained by using two-tailed Student’s *t* test.

## Results

### LNFPIII-NGC induces APC NFkB activation through the TLR4/MD2/CD14 complex

**Fig 1:**
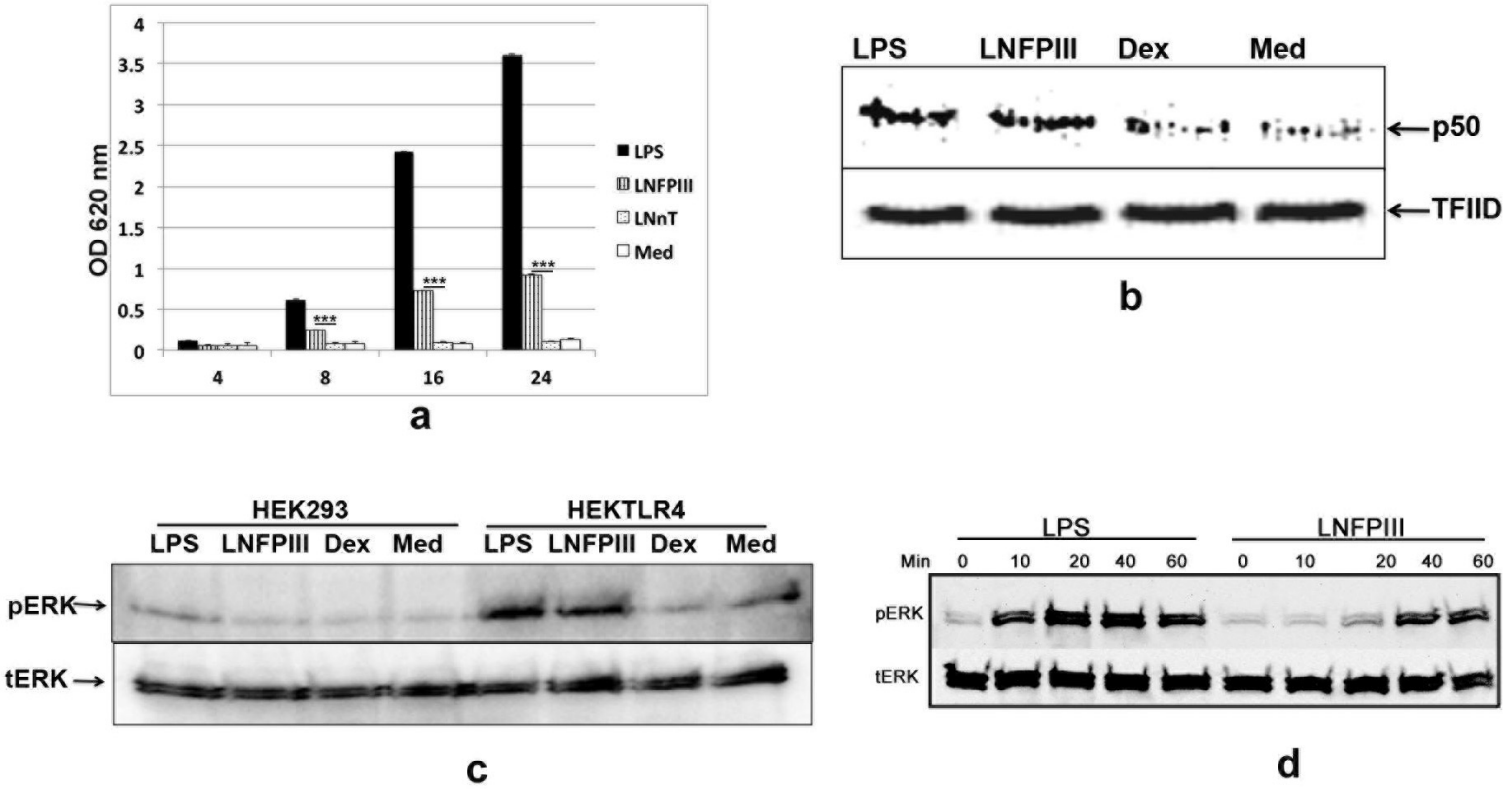
LNFPIII-NGC induces NFkB and ERK activation *via* the TLR4/CD14 receptor complex. a) HEKTLR4_AP (HEK293 cells stably transfected with TLR4/MD2/CD14 and alkaline phosphatase reporter gene cloned under the conserved NFkB binding site) cells were stimulated with LPS (100ng/ml), LNFPIII-NGC (50μg/ml), LNnT-NGC (50μg/ml) or media for various time points in hrs (as indicated) at 37˚C. The supernatants were collected and the relative amount of alkaline phosphatase was determined using colorimetric assay (OD at ~620nm). b) RAW 264.7 cells were stimulated with LPS (100ng/ml), LNFPIII-NGC (50ug/ml) and control dextran (50ug/ml) for 60 min at 37˚C. Nuclear extracts prepared and proteins separated on SDS-PAGE. Western blot of the same was probed with anti-p50 antibody and reprobed with TFIID antibody for loading control. c) HEK 293 cells and HEKTLR4 cells were stimulated with LPS (100ng/ml), LNFPIII-NGC (50ug/ml), control dextran (50ug/ml) or media alone for 60 min at 37˚C. Cell lysates were fractionated on SDS-PAGE and western blot probed for pERK induction. The same blot was reprobed for total ERK protein. d) RAW 264.7 cells were stimulated with LPS (100ng/ml) and LNFPIII-NGC (50ug/ml) for different time points (10, 20, 40 and 60 minutes) at 37˚C. Cell lysates were prepared and proteins were fractionated on SDS-PAGE. Western blot for the same was probed with pERK antibody and reprobed with total ERK antibody.

Previously, LNFPIII-NGC failed to drive DC2 maturation in dendritic cells lacking TLR4 or NFkB signaling [1,2]. To determine if the ability of LNFPIII-NGC to drive DC2 maturation is solely due to TLR4 or is associated with the receptor complex, we looked for NFkB activation using an alkaline phosphatase reporter assay in HEK 293 cells expressing TLR4/CD14/MD2 (HEKTLR4_AP), where the alkaline phosphatase gene is cloned downstream to the NFkB promoter. HEKTLR4 cells were incubated with LNFPIII-NGC for different time intervals (hrs) and then the level of alkaline phosphatase was measured in supernatants. We observed LNFPIII induced NFkB activation in HEKTLR4_AP cells in a time dependent manner (Fig 1A and 1B). In contrast, neo-glycoconjugates of LNnT, the identical tetrasaccharide backbone but lacking the fucose side chain of LNFPIII, failed to drive NFkB activation through the TLR4 receptor complex suggesting that the Le^x^ motif present in LNFPIII is essential for signaling through this receptor complex (Fig 1A). We then looked for the requirement of the TLR4 complex in LNFPIII-NGC activation of ERK in the MAPK pathway. Incubation of LNFPIII-NGC with HEKTLR4 cells induced phosphorylation of ERK, which was not detected in non-transfected HEK293 (Fig 1C). Further, comparison of LNFPIII-NGC and LPS activation of RAW 264.7 macrophages showed different ERK activation patterns. We stimulated cells for 10, 20, 40 and 60 minutes to observe the pERK induction pattern and found that LNFPIII-NGC drives late activation of ERK with peak phosphorylation at 60 mins, whereas LPS induced ERK activation as early as 20 mins (Fig 1D). For our studies we used 100ng/ml of LPS and 50ug/ml of LNFPIII-NGC as indicated in previous publications [4, 6].

### CD14 is required for LNFPIII-NGC induced ERK and NFkB activation

**Fig 2:**
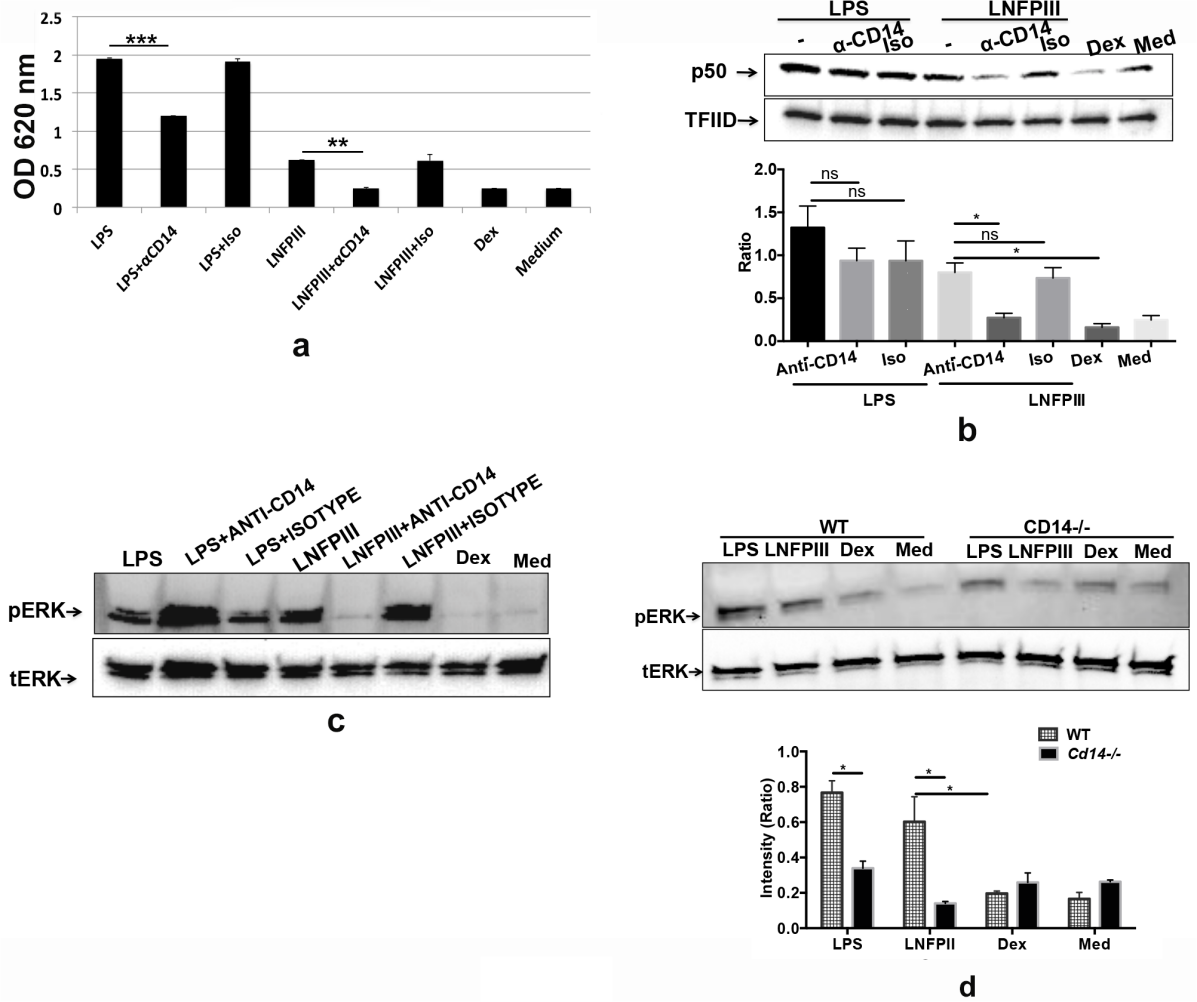
CD14 is required for LNFPIII-NGC induced ERK and NFκB activation. a) HEKTLR4_AP cells were pretreated with anti-CD14 antibody or isotype control for 90 mins at 37˚C followed by stimulation with LPS (100ng/ml), LNFPIII (50μg/ml), Dex (50μg/ml) or media control for 20–24 hrs at 37˚C. The supernatants were collected and levels of alkaline phosphatase were determined. Statistical significance was calculated using GraphPad student’s unpaired *t* test. b-c) RAW 264.7 cells were treated with anti-CD14 antibody or isotype control for 90 mins at 37°C followed by stimulation with LPS (100ng/ml), LNFPIII (50μg/ml), Dex (50μg/ml) or media alone for 60 min. The nuclear extracts or the whole cell lysate was then fractionated using SDS-PAGE and probed with antibody against NFκB subunit p50 (b) or phospho-ERK1/2 (c) and the blots were re-probed with anti-TFIID (b) or anti-total-ERK antibodies (c). Bar graph shows the ratio of intensities of the pERK and the control (tERK) bands. d) BMDCs from WT (C57BL/6J) or CD14-/- knock out (C57BL/6J) were stimulated with LPS (100ng/ml), LNFPIII (50μg/ml), Dex (50μg/ml) for 60 min. The whole cell lysate was then fractionated using SDS-PAGE and probed with anti-pERK antibody and the blot was re-probed with anti-total ERK antibody.

We next asked if the results are solely due to TLR4, or if CD14 plays a role in the signaling process, focusing on CD14 as co-receptor. Pre-treating HEK-TLR4 cells with neutralizing anti-CD14 antibody completely abolished the ability of LNFPIII-NGC to induce NFkB activation (Fig 2A). In regards to alternative activation and/or tolerance induction, NFkB subunit p50 accumulation has been shown to promote both of these responses, and earlier we demonstrated that LNFPIII-NGC influenced NFkB translocation via the p50 subunit[4, 13, 14]. Here we found that pretreatment of RAW macrophages with anti-CD14 antibody significantly reduced LNFPIII-NGC, but not LPS, induced nuclear translocation of NFkB subunit p50 (Fig 2B). ERK activation in LNFPIII-NGC stimulated RAW macrophages was significantly reduced in cells pretreated with anti-CD14 antibody, which had no impact on LPS induced ERK activation (Fig 2C). Similarly, LNFPIII-NGC induced ERK activation was abrogated in *Cd14* ^*-/-*^ BMDCs whereas the ability of LPS to induce ERK activation in these cells was not affected (Fig 2D). Taken together, these studies confirm a crucial role of CD14 in LNFPIII-NGC induced TLR4 dependent signaling, and clearly show major differences between LNFPIII-NGC and LPS activation of APCs via the TLR4/CD14 signaling complex.

### LNFPIII-NGC induces TLR4/CD14 mediated Raf-1 kinase activation

**Fig 3:**
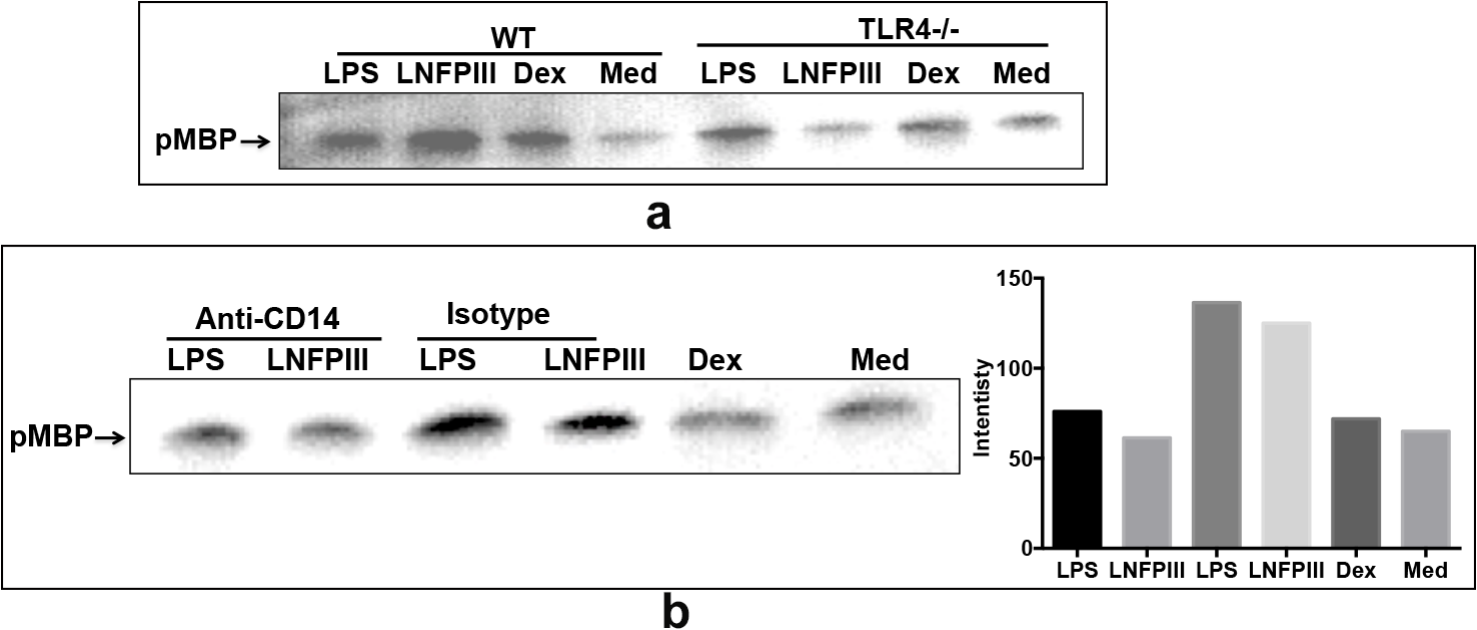
LNFPIII induces Raf-1 kinase activation *via* TLR4 and CD14 receptors. a) BMDCs from *wt* and *Tlr4* ^*-/-*^ mice were stimulated with LPS (100ng/ml), LNFPIII-Dex (50μg/ml), Dex (50μg/ml) or nothing for 15 mins. Raf-1 kinase activity was carried out as described in materials and methods. The reaction mixtures were fractionated using SDS-PAGE and western blot performed with anti-phospho MBP antibody. b) RAW264.7 macrophages were pre-treated with anti-CD14 antibody and isotype control followed by stimulation with LPS (100ng/ml), LNFPIII-NGC (50μg/ml), Dex (50μg/ml) or nothing for 15 mins, processed for Raf-1 kinase assay and western blot analysis was performed using anti-phospho-MBP antibody.

An earlier study suggested that Lewis^X^ conjugated to polyacrylamide, was able to activate human dendritic cells, inducing a signaling cascade that involved Raf-1 kinase[12]. Therefore we evaluated the ability of LNFPIII-NGC to induce Raf-1 kinase activity in RAW 264.7 macrophages. Raf-1 kinase activity was assayed *in-vitro* by measuring the phosphorylation intensity of Myelin basic protein (MBP). LNFPIII-NGC activated macrophages or BMDCs showed enhanced Raf-1 kinase activity in both a TLR4 and CD14 dependent manner (Fig 3A and 3B). BMDCs isolated from WT and *Tlr4* ^*-/-*^ mice were treated with different TLR4 agonists. Both LPS and LNFPIII-NGC induced an enhanced level of Raf-1 kinase activity, which was completely absent in *Tlr4* ^*-/-*^ BMDCs (Fig 3A). Similar to our findings with ERk and NFkB, when we treated RAW 264.7 cells with LFPIII-NGC in the presence of anti-CD14 antibody, we found significantly abrogated Raf1 kinase activation compared to isotype control treated cells. LPS induced Raf-1 kinase activity was only partially affected by blocking the CD14 receptor. Band signal intensity from western blots were analyzed to confirm the findings (Fig 3B).

### LNFPIII-NGC induced ERK activation is *via* the TLR4/CD14-syk-tpl2-Ras-Raf1-MEK pathway

**Fig 4:**
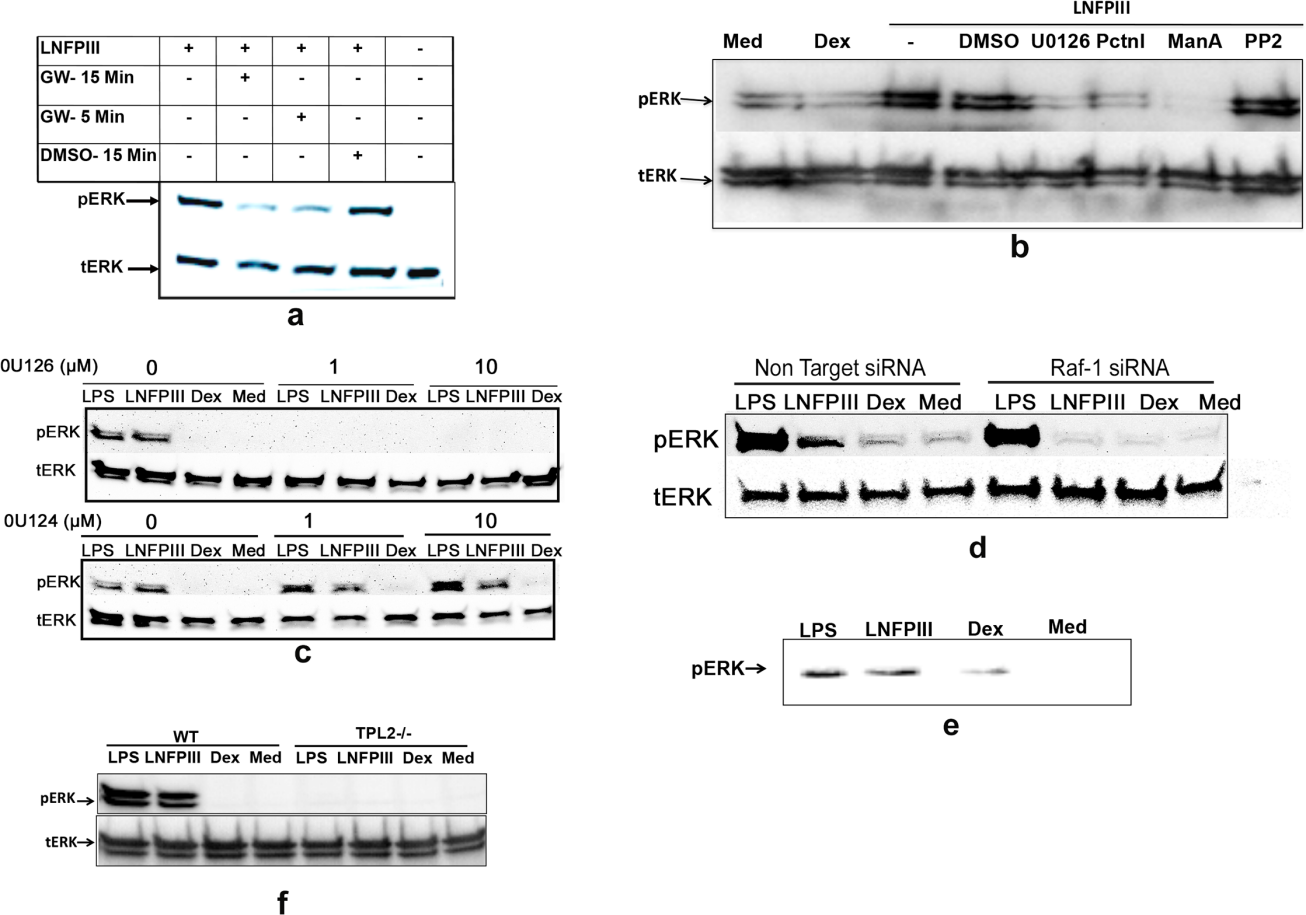
LNFPIII induces ERK activation *via* the Syk-Ras-TPL2-Raf-MEK pathway. a-b) RAW 264.7 cells were pretreated with Raf-1 inhibitors against Raf-1 (GW5074) or DMSO (a) for 5 and 15 mins and MEK (U0126), syk (piceatannol), Ras (ManA) and src (PP2) (b) for 60 mins followed by stimulation with LNFPIII-NGC (50μg/ml), Dex (50μg/ml) and media alone for 60 min at 37°C. Western blot was performed using anti-pERK antibody and the blot was re-probed with anti-total ERK antibody. Bar graph shows the ratio of intensities of the pERK and the control (tERK) bands. c) RAW264.7 cells were pre-treated with 0 μM, 1 μM or 10 μM of U0126 or U0124 inhibitor followed by stimulation with LPS (100ng/ml), LNFPIII-Dex (50μg/ml), Dex (50μg/ml) or nothing for 60 mins at 37°C. Whole cell lysate was then fractionated using SDS-PAGE and probed with anti-pERK antibody and the blot re-probed with anti-total ERK antibody. d) BMDCs were transfected with siRNA against Raf-1 or non-target control, followed by stimulation with LPS (100ng/ml), LNFPIII-Dex (50μg/ml), Dex (50μg/ml) or nothing for 60 mins at 37°C. Cell lysates were processed for western blot as described above. e) RAW 264.7 cells were pre-treated with 0 μM, 1 μM or 10 μM of U0126 or U0124 stimulated with LPS (100ng/ml), LNFPIII-Dex (50μg/ml), Dex (50μg/ml) or nothing for 15 mins at 37°C. Cell lysates were incubated with anti-Raf-1 antibody. The pulled down mixtures were fractionated using SDS-PAGE and probed with anti-phospho ERK antibody. f) BMDMs from *wt* and *Tpl2-/-* mice were treated with LNFPIII-NGC (P3), Dex and Media alone for 60 mins at 37°C. Whole cell lysate was then fractionated using SDS-PAGE and probed with anti-pERK antibody and the blot re-probed with anti-total ERK antibody.

LNFPIII-NGC induces differential activation of MAPK by selectively driving ERK phosphorylation, and not JNK or p38[6]. Having demonstrated that LNFPIII-NGC drives TLR4/CD14 dependent Raf-1 activation we next tested roles of signaling molecules upstream or downstream to Raf-1 in LNFPIII-NGC driven ERK activation. Pretreatment of RAW 264.7 cells, with an inhibitor of Raf-1 kinase (GW5074), followed by LNFPIII-NGC stimulation, resulted in significant inhibition in LNFPIII-NGC induced ERK activation (Fig 4A). We expanded the analysis to examine pharmacological inhibitors against Src family kinase, Syk tyrosine kinase, Ras GTPase and MEK to further validate that LNFPIII-NGC induces ERK activation through the Ras-Raf-MEK classical MAPK pathway. We observed that LNFPIII-NGC activation of ERK was inhibited in cells pre-treated with the inhibitors Piceatannol (Syk inhibitor), Manumycin A (inhibits Ras activation) and U0126 (MEK inhibitor) whereas no effect was seen in cells pre-treated with PP2 (Src family kinase inhibitor) (Fig 4B) suggesting that LNFPIII-NGC induces Syk, Ras and MEK dependent and Src independent ERK activation. To study dose dependency and rule out any cytotoxity the inhibitors might have at higher concentrations, we used 1μM and 10μM concentrations of U0126 MEK inhibitor, and found that both were efficient in blocking ERK phosphorylation in response to LNFPIII-NGC or LPS. Use of the inactive analog (U0124) of U0126 did not prevent LNFPIII-NGC or LPS induced ERK activation (Fig 4C). To validate the data obtained using chemical inhibitors, we utilized an SiRNA approach to knock down the expression of Raf1 kinase in BMDCs and found that LNFPIII-NGC failed to activate ERK phosphorylation in BMDCs transfected with anti-Raf1 SiRNA compared to the non-Target control (Fig 4D). We further demonstrated an association of pERK with Raf-1 using immune pull-down assays to confirm that LNFPIII-NGC signals through the classical Ras-Raf-1-ERK MAPK pathway (Fig 4E). Tumor progression locus 2 (TLP2) is a MAP3K molecule essential for TLR4 signaling in activating ERK in bone marrow derived macrophages. TPL2 function is partially compensated by Raf-1 in murine bone marrow derived dendritic cells[15]. We tested for a role of TPL2 in LNFPIII-NGC induced ERK signaling with bone marrow derived macrophages (BMDMs) from *Tpl2* ^*-/-*^ and WT mice. Compared to WT BMDMs, which had enhanced ERK activation after stimulation with LNFPIII-NGC, LNFPIII-NGC failed to induce ERK activation in *Tpl2* ^*-/-*^ BMDMs (Fig 4F). Over all the data suggest that LNFPIII-NGC induces a TLR4/CD14 dependent Syk-Ras-Raf1-MEK, TPL2 dependent signaling cascade in both RAW 264.7 macrophages and BMDCs.

### LNFPIII-NGC co-localizes with CD14 and mediates surface signaling

**Fig 5:**
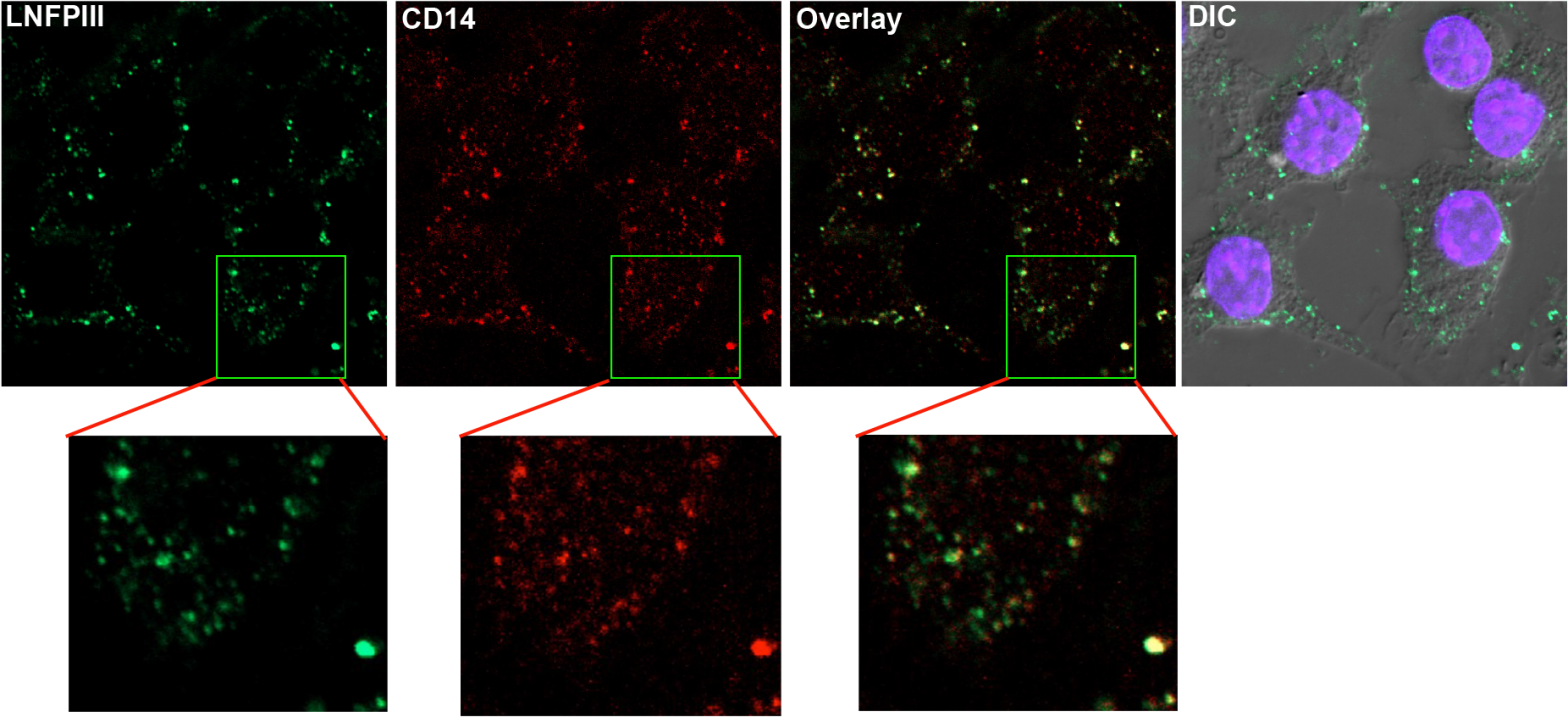
LNFPIII-NGC co-localizes with CD14. LNFPIII-NGC was incubated with RAW 264.7 cells for 20 min at 37°C. Cells were fixed and double stained for LNFPIII-NGC (green) and CD14 (red). Confocal images were obtained using a Nikon A1R confocal microscope under 60X objective.

Having demonstrated that LNFPIII-NGC drives Ras-Raf-ERK signaling via CD14, we asked if LNFPIII-NGC co-localizes with CD14 on the surface of APCs. To define LNFPIII-NGC localization, we probed using a monoclonal IgM antibody (E.5) recognizing LNFPIII-NGC[11]. We observed that LNFPIII-NGC co-localized with CD14 in bone marrow derived dendritic cells (BMDCs) (Fig 5). We observed no significant uptake or surface binding of texas-red labeled dextran (carrier molecule for LNFPIII), confirming that CD14 binding was LNFPIII specific. Using the LNFPIII specific monoclonal antibody E.5, we also demonstrated LNFPIII-NGC co-localization with TLR4 on both RAW 264.7 and BMDCs suggesting that LNFPIII-NGC binds to the TLR4/CD14 receptor complex in driving an anti-inflammatory signaling cascade (S2 Fig).

### LNFPIII-NGC stimulation induces secretion of anti-inflammatory chemokines and cytokines

**Fig 6:**
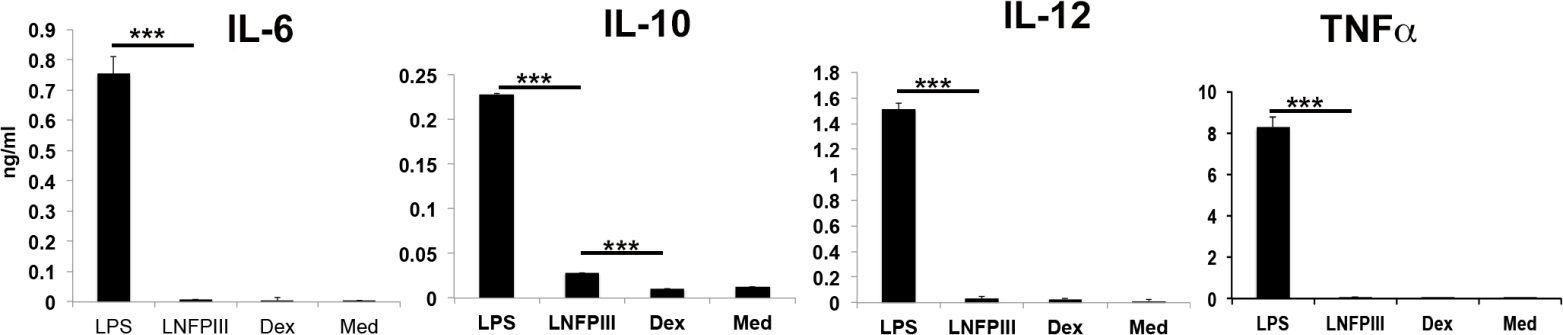
LNFPIII induces biased chemokine/cytokine production. BMDCs were stimulated with LPS (100ng/ml), LNFPIII-NGC (50μg/ml), Dex (50μg/ml) or nothing for 24 hrs at 37°C. Supernatant from each well was collected and cytokine levels (as mentioned) measured using ELISA. Statistical significance was determined using GraphPad student’s unpaired *t* test method.

**Fig 7:**
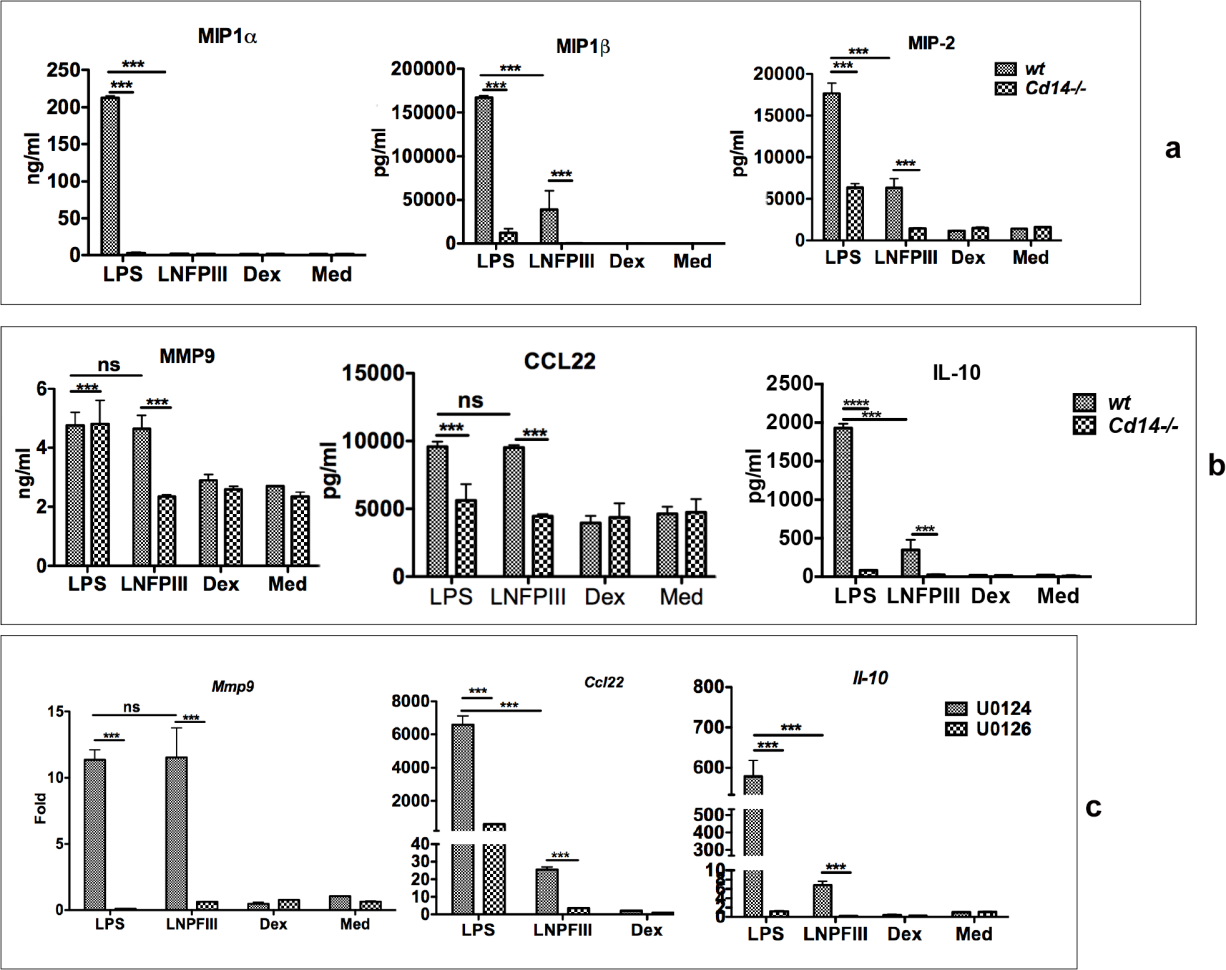
LNFPIII-NGC drives anti-inflammatory chemokine/cytokine production. a-b) BMDCs from WT and CD14-/- mice were stimulated with LPS (100ng/ml), LNFPIII-NGC (50μg/ml), Dex (50μg/ml) or nothing for 24 hrs at 37C. Supernatant from each well was collected and sent for RBM analysis. Statistical analysis was performed using GraphPad student’s unpaired *t* test method. c) BMDMs were treated with MEK1/2 inhibitor, U0126 or control inhibitor (U0124) for 30 minutes followed by stimulation with LPS (100ng/ml), LNFPIII-NGC (50μg/ml), Dex (50μg/ml) or nothing for 4 Hrs at 37°C. Cells were harvested, total RNA isolated and qRT-PCR was performed using respective primers (as mentioned). Statistical significance was measured using GraphPad student’s unpaired *t* test method.

Although LNFPIII-NGC activation of APCs occurs via a TLR4/CD14 signaling pathway, LNFPIII stimulated APCs preferentially drive CD4+ T cells to produce the Th2 cytokines IL-4, IL-13 and also IL-10 *in-vivo*[16–19]. Here we broadened our analysis of cytokines and chemokines produced by LPS or LNFPIII-NGC stimulated BMDCs and RAW 264.7 cells *in vitro*. As expected, we observed an increase in level of IL-10 production from LNFPIII-NGC stimulated cells (Fig 6). However, unlike LPS, LNFPIII-NGC did not induce production of IL-6, IL-12 or TNF-α in these cells (Fig 6) in spite of enhanced NFkB and ERK activation (Figs 1 and 2). These results suggest that unlike LPS, LNFPIII-NGC selectively regulates IL-10 production. In order to look at other cellular factors that would facilitate the induction of LNFPIII-NGC mediated Th2 or anti-inflammatory responses, we performed Rules Based Medicine (RBM) analysis on supernatants from LNFPIII-NGC treated BMDCs. Interestingly, in response to LNFPIII-NGC we observed elevated production of macrophage driving chemokine (MDC/CCL22) and matrix metalloproteinase (Mmp9) compared to BMDCs stimulated with LPS (Fig 7B). MDC/CCL22 is an important chemokine, which favors recruitment of CD4^+^ T-cells that mediate Th2 type responses. Similarly, MMP9 is expressed in livers of *S*. *mansoni* infected mice in response to the Th2 cytokine IL-13 [20]. Production of IL-10 was also observed in BMDCs, which was dependent on CD14 as CD14 deficient BMDCs showed reduced secretion of IL-10 in response to LNFPIII-NGC. (Fig 7B). In addition, RBM analysis showed significantly lower amounts of “Th1” or “pro-inflammatory” chemokines (MIP-2, MIP-1ß and MIP-α) from LNFPIII-NGC stimulated cells compared to LPS stimulation (Fig 7A). Interestingly, LNFPIII-NGC induced secretion of both MDC (CCL2 and Mmp 9 were completely abrogated in CD14-/- BMDCs, in contrast to the limited reduction of these responses in LPS stimulated CD14-/- BMDCs (Fig 7B). These findings show that unlike LPS, LNFPIII-NGC induces CD14 dependent production of chemokines that likely help in driving LNFPIII-NGC induced Th2 immune responses.

Next we determined if MAPK signaling was responsible for the production of anti- inflammatory chemokines or cytokines in response to LNFPIII-NGC stimulation. For this, macrophages were incubated with highly selective inhibitors (U0126) for MEK1 and MEK2 kinases or control inhibitor (U0124) followed by stimulation with LNFPIII-NGC or LPS. Post-stimulation, we measured the expression of *Ccl22 (Mdc)*, *Mmp9* and *Il-10* at the mRNA level. As shown in Fig 7C macrophages incubated with U0126 had significantly reduced expression of chemokines (*CCl22* and *Mmp9)* as well as *Il-10* compared to macrophages incubated with the control inhibitor, U0124. This demonstrates that LNFPIII-NGC drives CCl22, Mmp9 and Il-10 via MEK1/MEK2 pathways.

## Discussion

Conjugates of the human milk sugar LNFPIII function *in vivo* as anti-inflammatory and therapeutic agent for murine experimental autoimmune encephalomyelitis, psoriasis, as an anti-rejection agent in organ transplant and in obesity induced metabolic disease[1, 2, 21, 22]. LNFPIII-NGC functions as anti-inflammatory therapeutic by activating and inducing maturation of antigen presenting cells that are anti-inflammatory[2]. Interestingly, LNFPIII-NGC activation of APCs requires signaling *via* the TLR4 complex, as LNFPIII-NGC stimulated TLR4-/- DCs were unable to drive CD4^+^ T cells to produce IL-4[6]. The inability to drive CD4+ T cells to Th2-type was associated with defects in activation of MAPK and NFkB signaling pathways in TLR4-/- BMDCs, as measured by activation of ERK1/2 and nuclear translocation of NFkB p50[4, 6]. TLR4 functions in a complex with MD2 and CD14 as co-receptors to trigger signaling cascades for APC activation. CD14 is the first pattern recognition receptor described, and in addition to TLR4, has been shown to assist TLRs-3, -7 and 9 in mediating immune activation[7–9].

Thus, a key question is how can bacterial LPS and LNFPIII-NGC both signal through the same TLR4/MD2/CD14 receptor complex, yet induce disparate APC signaling and maturation pathways? We pursued this question by asking if LNFPIII-NGC induced activation of APCs was dependent on TLR4 mediated signaling cascades alone or in association with other innate immune receptors. In the study reported here, we confirmed, using TLR4-MD2-CD14 transfected HEK293 NFkB reporter cells that the TLR4 receptor complex (TLR4-MD2-CD14) was sufficient to drive LNFPIII-NGC mediated NFκB and MAPK activation. However, we believe the major finding from our study was to demonstrate the requirement of the TLR4 co-receptor CD14 in triggering LNFPIII-NGC induced signaling cascades. In this regard, we demonstrated the required role of CD14 using several different methods and cell types, including CD14 deficient BMDCs, neutralizing antibody to CD14, and over expression of CD14/TLR4 complex in HEK293 cells. The requirement of CD14 for LNFPIII-NGC induced signaling in APCs was supported by our observation that LNFPIII-NGC co-localizes with CD14 on and in APCs, suggesting that CD14 is an important innate receptor for LNFPIII-NGC induced signaling (Fig 1, Fig 2 and Fig 5). The critical role of CD14 in LNFPIII-NGC induced signaling makes sense as we recently demonstrated that CD14 regulates alternative activation of macrophages and the intensity of CD4^+^ Th2-type responses in schistosome infected mice [23]. Schistosome glycans, including Lewis^x^, are required for induction of CD4+ Th2-type responses, and helminth glycans and LNFPIII-NGC containing Lewis^x^ motif induce alternative activation of APCs.

The ability of complex carbohydrates to interact with the TLR4 signaling complex has been evaluated in other studies on helminth glycan modulation of APC activity. Schistosome glycans were shown to interact with TLR4 and C-type lectin receptors (DC-SIGN) to modulate APC functions[24]. Observations with Lewis^x^ conjugate activated human cells noted activation of Raf-1 kinase through DC-SIGN[25]. Further, conjugates of Lewis^x^, a glycan expressed by helminthes and humans, were shown to interact with DC-SIGN, leading to suppression of LPS induced IL-6 and IL-12 production *via* Raf-1 kinase activation[26, 27]. In this regard, a recent study by Koning et al [28] demonstrated that Lewis^x^ on the human milk glycoprotein MUC1, prevented pathogen interaction with innate immune cells by blocking interaction with DC-SIGN.

In this study we show that LNFPIII-NGCs drives Raf-1 kinase activation *via* the TLR4-CD14 complex, mediating classical Ras-Raf-MEK activation. Previous studies demonstrated that Lewis^X^ conjugates, glyco-lipids or glyco-proteins differentially activate APCs, via a process that required co-operative action of both DC-SIGN and TLR4[24]. In a recent study by Terrazas et al, activation of Raf1 in DCs was shown to play an important role in induction of Th2 responses [29]. The authors found that DCs treated with excreted/secreted product of *Taenia crassiceps* (TcES) abrogate TLR induced secretion of TNFα and IL-12 cytokines, but drive strong Th2 responses in a cRaf (Raf1) dependent manner [29]. Cellular activation was dependent on recognition of TcES not only by TLR2, but also by the C-type lectins mannose receptor (MR) and MGL. To determine if DC-SIGN influences TLR4 mediated LNFPIII-NGC signaling, we over-expressed SIGNR-1, SIGNR-3 and SIGNR-5 (human homologues of DC-SIGN receptor) in HEK293 cells and did not observe any differences on LNFPIII-NGC-TLR4 signaling in these cells (S1 Fig), although LNFPIII-NGC showed a strong interaction with SIGNR-1Differences in levels of cellular activation by glycan containing ligands, including Lewis^x^ motif and LNFPIII-NGC, may be due to LNFPIII being a pentasaccharide, which when conjugated to a carrier, likely has greater “rotational flexibility” off of the carrier molecule to interact with cellular receptors compared to Lewis^x^ trisaccharide conjugates. Alternatively, it could be as simple as density of glycan residues/carrier molecule, with the Lewis^x^-polyacrylamide constructs having minimally three times the density of glycan/carrier as the LNFPIII-NGC conjugates.

The ability of LNFPIII-NGC to activate APCs and induce their maturation to anti-inflammatory phenotype, has led to numerous applications of LNFPIII-NGC as preventative or therapeutic for diseases that are inflammation based. For example, in the murine High-Fat diet induced model of obesity, treatment of mice with LNFPIII-NGC alleviated liver hepatosteatosis and improved insulin sensitivity in part, *via* a macrophage IL-10 dependent mechanism[2]. Here, we demonstrated that LNFPIII-NGC induction of IL-10 and the Th2 favoring molecules MDC and MMP9 was dependent on the MAPK pathway as inhibition of MEK1/2 kinases led to abrogation of LNFPIII induced *Il-10*, *Mmp9* or *Mdc* expression in bone marrow derived macrophages (Fig 7C). While, both LPS and LNFPIII-NGC signal in part via the TLR4-CD14 complex, there are key differences in the signaling cascades these two different ligands induce. For example, the peak activation of ERK1/2 in response to LNFPIII-NGC was observed at 60 mins, whereas in response to LPS, ERK1/2 activation could be seen as early as 20 mins with peak activation at 40 mins post incubation. Further, CD14 was required for LNFPIII-NGC but had only limited influence on LPS induced ERK activation (Fig 2C and 2D) (Fig 1). These observations suggest that differential activation in ERK1/2 mediated signaling is crucial for LNFPIII-NGC induction of anti-inflammatory responses. This is in agreement with previous findings where sustained ERK1/2 activation was associated with programming of DCs to induce anti-inflammatory or Th2 responses *via* ERK dependent c-Fos transcription factor[30, 31]. Activation of ERK in response to LNFPIII was also dependent on TPL2 (MAP3K8), which has been implicated in promoting anti-inflammatory responses *via* ERK-c-Fos MAPK pathway[32]. TPL2 has been shown to promote alternative activation and block classical activation of macrophages by regulating the expression of AA markers, Ym1 and RELMα, and M1 marker, *Nos2* or nitric oxide (NO) production[33, 34]. Overall, our finding that LNFPIII-NGC utilizes Syk-Ras-Raf-TPL2-MEK-ERK pathway to modulate APC function leading to induction of anti-inflammatory responses is in agreement with previously reported studies on alternative or M2 activation.

We previously reported that LNFPIII-NGC activation of APCs altered the kinetics of NFkB translocation compared to levels in LPS activated cells[4]. We further investigated kinetics of NFkB translocation in the context of requiring CD14 in LNFPIII-NGC activated vs LPS activated APCs. In addition, to partially examine glycan structural specificity for CD14, we evaluated a neo-glycoconjugate comprised of the tetrasaccharide LNnT, conjugated to the 40 kDa dextran via the same process and in the same lab that LNFPIII-NGC is prepared. LNnT was chosen as it has the identical tetrasaccharide backbone as LNFPIII, but lacks the alpha 1–3 linked fucose of LNFPIII. Importantly, we found using a HEK293 reporter cell assay that both LPS and LNFPIII-NGC drove NFkB translocation in a TLR4-CD14 mediated activation step, but that LNnT-NGC did not, suggesting that the alpha 1–3 linked fucose on LNFPIII is critical for this process. This makes sense as previous studies have demonstrated that helminth expressed Lewis^x^, which has an alpha 1–3 linked fucose, activates APCs in a process that uses TLR4 and C-type lectins[25]. Identical to what we observed for activation of ERK, neutralization of, or deficiency of CD14, abrogated LNFPIII-NGC translocation of NFkB to a greater extent than was seen for LPS stimulated cells (Fig 2A and 2B). Interestingly, the absence of CD14 led to almost complete abrogation of NFkB p50 subunit translocation whereas this was largely unaffected in LPS stimulated cells. This is interesting as accumulation of p50 in LPS activated cells is thought to be regulatory, and inhibit the cells prolonged response to LPS[14]. Further, accumulation of p50 is thought to play a role in macrophage phenotype plasticity from classically activated to alternatively activated[13]. Overall, these studies suggest that s key structural element of LNFPII-NGC to induce TLR4-CD14 activation of APCs is the alpha 1–3 linked fucose. Further, we observed that CD14 deficiency impacts LNFPIII-NGC induced activation greater than LPS activated cells, specifically for the p50 subunit of NFkB. Whether or not structurally similar glycans having alpha 1–2, alpha 1–4 or beta 1–3 linked fucose, as found in LNFPI, LNFPII and LNFPV respectively, can activate APCs via TLR4-CD14 needs to be determined.

This observation on the requirement of the alpha 1–3 linked fucose for binding to and activation of APCs leading to altered NFkB kinetics is interesting in light of the normal events that occur during microbial infection. Neutrophils are generally the first cells to arrive at the site of infection, where they become activated. An inability to control neutrophil recruitment and activation often results in damage to surrounding tissue. Macrophages are normally the second cell type to arrive at the site of infection. Could macrophages be one of the cells that “regulate” neutrophil activation/recruitment? Possibly, neutrophils express glycans such as Lewis^x^[35–37]. It is tempting to hypothesize that activated neutrophils upregulate surface expression of Lewis^x^ and perhaps other glycans, that can then activate incoming macrophages and drive them to an alternative maturation and thus, anti-inflammatory phenotype, to minimize surrounding tissue damage.

**Fig 8:**
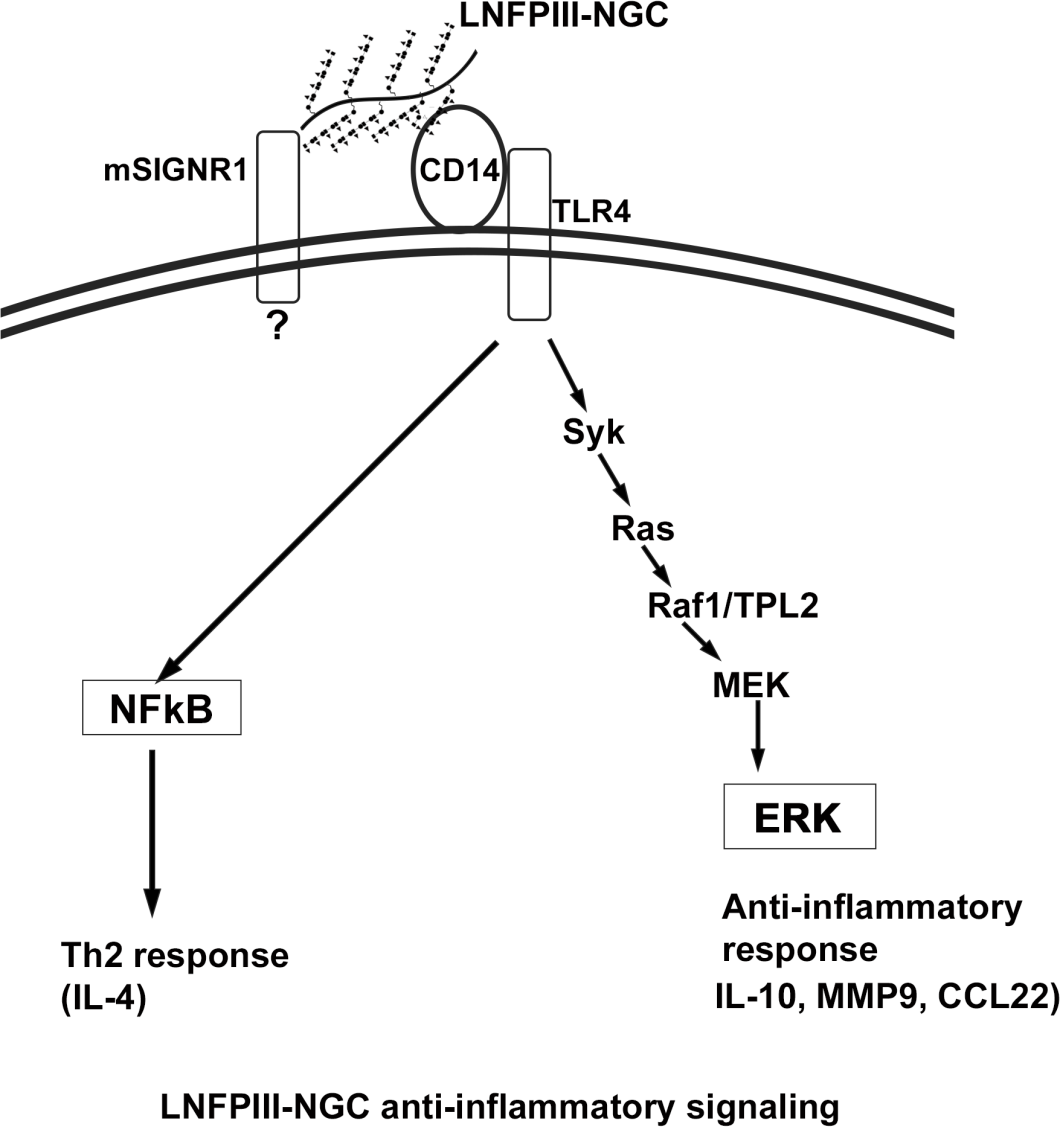
Model depicts the possible signaling pathways triggered by LNGPIII-NGC to drive anti-inflammatory responses in APCs.

Beyond the signaling cascade, we asked if LNFPIII-NGC activation of APCs induced cytokines and/or chemokines known to bias immune activation. To test this we performed rules based medicine (RBM) analysis and found various chemokines produced in response to LNFPIII-NGC stimulation. Compared to LPS, LNFPIII-NGCs induced significantly less production of pro-inflammatory chemokines MIP1-α, MIP1-β and RANTES in BMDCs. However, LNFPIII-NGC induced equivalent production of “Th2 or anti-inflammatory” favoring chemokines, MDC and matrix metalloproteinase 9 (MMP9) in DCs, which required CD14 and activation of ERK1/2 pathway (Fig 6). Over all, the data suggest that LNFPIII induces a biased “anti-inflammatory” response by activation of ERK1/2 *via* CD14/TLR4-syk-Raf-TPL2-MEK pathway that promotes production of anti-inflammatory chemokine/cytokines in APCs in a novel receptor/signaling cascade (Fig 8). The signaling cascade identified in this study may be explored in future studies evaluating the therapeutic activity of LNFPIII-NGCs. Understanding how this signaling cascade drives APCs towards anti-inflammatory is a key step towards development of new anti-inflammatory molecules. Further studies will determine if lack of any of the signaling molecules alters the ability of LNFPIII-NGC to function as therapeutic for pro-inflammatory and/or metabolic diseases.

## Supporting Information

S1 Fig No effect of C-type lectin receptors SIGNR-1, SIGNR-3, SIGN-5 on LNFPIII-NGC triggered TLR4/CD14 signaling. HEKTLR4_AP cells transfected with plasmids expressing C-type lectin receptors SIGNR-1, SIGNR-3 and SIGNR-5. Cells were stimulated with LNFPIII-NGC (50μg/ml), dextran carrier (50μg/ml) or media alone and incubated at 37°C for 24 hrs. The supernatants were collected and the relative amount of alkaline phosphatase (to measure NFκB activity) was determined using colorimetric assay (OD at ~620nm).
(TIFF)

S2 Fig LNFPIII-NGC co-localizes with TLR4. LNFPIII-NGC was incubated with RAW 264.7 cells for 20 min at 37°C. Cells were fixed and double stained for LNFPIII-NGC (green) and TLR4 (red). Confocal images were obtained using a Nikon A1R confocal microscope under 60X objective.
(TIFF)
