# Supplementary figures and images for: A Neoglycoconjugate Containing the Human Milk Sugar LNFPIII Drives Anti-Inflammatory Activation of Antigen Presenting Cells in a CD14 Dependent Pathway
Source: PLoS One. 2015 Sep 4;10(9):e0137495. doi: 10.1371/journal.pone.0137495 (PMC4560409; doi:10.1371/journal.pone.0137495)

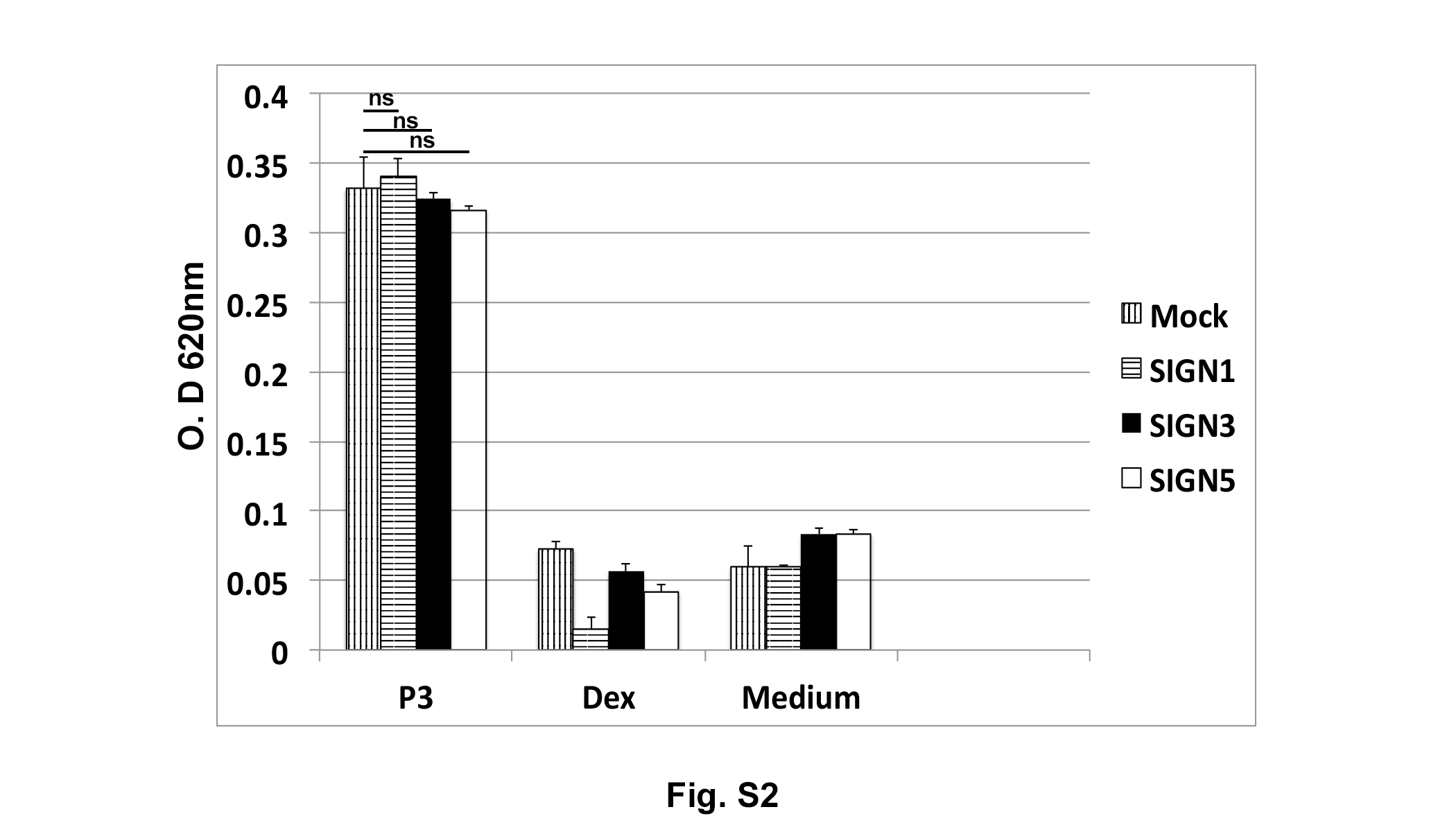

Supplement: S1 Fig — HEKTLR4_AP cells transfected with plasmids expressing C-type lectin receptors SIGNR-1, SIGNR-3 and SIGNR-5. Cells were stimulated with LNFPIII-NGC (50μg/ml), dextran carrier (50μg/ml) or media alone and incubated at 37°C for 24 hrs. The supernatants were collected and the relative amount of alkaline phosphatase (to measure NFκB activity) was determined using colorimetric assay (OD at ~620nm). (TIFF) [file pone.0137495.s001.tiff]

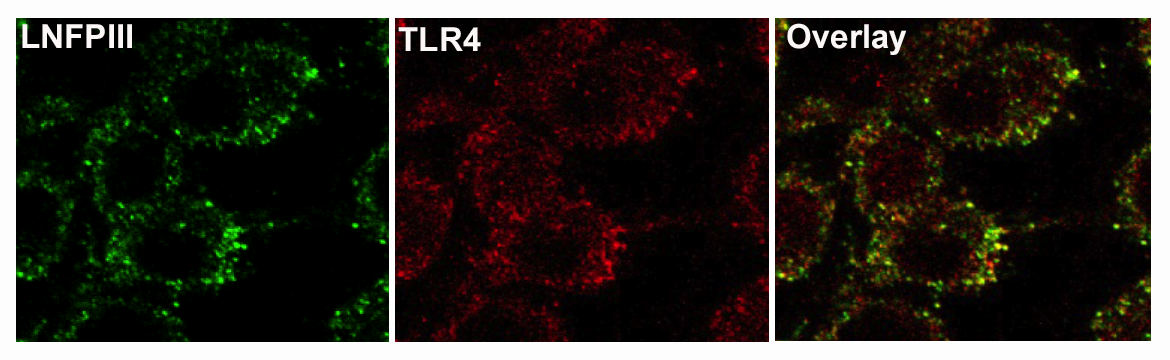

Supplement: S2 Fig — LNFPIII-NGC was incubated with RAW 264.7 cells for 20 min at 37°C. Cells were fixed and double stained for LNFPIII-NGC (green) and TLR4 (red). Confocal images were obtained using a Nikon A1R confocal microscope under 60X objective. (TIFF) [file pone.0137495.s002.tiff]
